# Prevalence, antimicrobial susceptibility profile, and associated risk factors of uropathogenic *Escherichia coli* among pregnant women attending Dr. Sumait Hospital Mogadishu, Somalia

**DOI:** 10.3389/fpubh.2023.1203913

**Published:** 2024-01-24

**Authors:** Fartun Yasin Mohamed, Hassan Abdullahi Dahie, Jamal Hassan Mohamoud, Mohamed Hussein Adam, Hassan Mohamud Dirie

**Affiliations:** ^1^Departments Microbiology and Medical Laboratory Sciences, Faculty of Medicine and Health Sciences, SIMAD University, Mogadishu, Somalia; ^2^SOS College of Health Science, SOS Children’s Villages, Mogadishu, Somalia; ^3^Department of Public Health, Faculty of Medicine and Health Sciences, SIMAD University, Mogadishu, Somalia

**Keywords:** pregnant women, *Escherichia coli*, antimicrobial, prevalence of *Escherichia coli*, urinary tract infections

## Abstract

**Background:**

Uropathogenic *Escherichia coli* (UPEC) is a strain of *E. coli* commonly associated with urinary tract infections. In addition, antibiotic resistance in UPEC is one of the most significant health problems. This study was conducted to determine the prevalence, antimicrobial resistance, and factors linked to uropathogenic *Escherichia coli* (UPEC) in pregnant women.

**Methods:**

This cross-sectional study was conducted within a hospital setting between August 2022 and December 2022. Using consecutive convenient sampling, the research enrolled 220 pregnant women. The urine samples obtained from these women were cultured on MacConkey and blood agar and incubated at 37°C overnight, followed by sub-culturing on Mueller Hinton media. Bacterial identification involved Gram staining and biochemical characterization (TSI, indole, citrate, methyl red, urea agar, and motility tests). Conversely, susceptibility tests were performed using the Kirby–Bauer disk diffusion method. A binary logistic regression model and analysis of odds ratios (ORs) were employed to evaluate the risk factors associated with *E. coli* infection, and statistical significance was attributed to *p*-values of ≤0.05.

**Results:**

Out of the 220 urine samples examined, 42 (19%) exhibited a positive culture, indicating an *E. coli* infection in pregnant women. Our analysis revealed that income, gestational age, and history of UTIs were identified as risk factors associated with *E. coli* infection. Most *E. coli* isolates demonstrated sensitivity to amikacin (100%), nitrofurantoin (85.7%), amoxicillin/clavulanic acid, and meropenem (83.3%).

**Conclusion:**

The prevalence of *E. coli* was remarkable. It could be recommended that pregnant women in antenatal care have routine culture and antimicrobial susceptibility tests to prevent transmission of resistant pathogens and complications in both pregnant mothers and the unborn baby.

## 1 Introduction

A urinary tract infection (UTI) occurs when a pathogen invades and multiplies within the urinary system, disrupting kidney and urinary function, and may present as symptomatic or asymptomatic bacteriuria (1). Uropathogenic *E. coli* (UPEC) is the predominant agent leading to urinary tract infections (UTIs), and it is widely recognized that pregnant women are at a higher risk of experiencing UTIs (2). Urinary tract infections are highly prevalent in pregnancy and can pose significant risks for both the mother and the unborn child (3). Due to the anatomical, physiological, and functional changes brought on by pregnancy, the urinary system is frequently susceptible to urinary tract infections (UTIs), which are a result of bacteria entering the urinary bladder (4). Globally, 13%–33% of pregnant women have UTIs, with 1%–18% experiencing symptoms and 2%–10% asymptomatic (5). Urinary tract infections (UTIs) are commonly seen in both community and hospital settings globally. Uropathogenic *Escherichia coli* is the causative agent in up to 90% of UTIs acquired in the community and 50% of those acquired in healthcare facilities (1). Several factors, such as multiple pregnancies, age, a history of urinary tract infections, diabetes, anatomical abnormalities in the urinary tract, inadequate personal hygiene, and socioeconomic status, influence the occurrence of bacteriuria during pregnancy (6).

*Escherichia coli* is the most prevalent pathogen, causing between 75% and 90% of bacteriuria in pregnant women (7–9). Additionally, pregnant women are considered vulnerable hosts for UTIs due to the physiological changes associated with pregnancy, leading to compromised immunity (4). Long-term untreated UPEC infection has also been known to be associated with pregnancy complications such as eclampsia, low birth weight, and preterm birth (10). Therefore, early UTI diagnosis, appropriate management, and a suitable therapeutic and preventive approach are crucial to avoiding pregnancy complications (11). The bacterial population in the human body can be significantly influenced by brief antibiotic exposure, leading to the development of resistant pathogens and symbiotic organisms. Antibiotic resistance may result in heightened patient morbidity, prolonged treatment, increased hospitalization, and reliance on broad-spectrum antibiotics (12). The emergence of drug-resistant strains of UPEC increases the serious threat to global health (13). The development of antimicrobial resistance in uropathogenic *Escherichia coli* (UPEC) and the emergence of multi-drug resistance (MDR) UPEC in recent years pose a clinical challenge, especially in women experiencing recurrent UTIs (14). The resistance patterns of bacteria can differ based on geographical location and time, highlighting the importance of regular testing for antibiotic resistance. *E. coli* strains are primary contributors to severe bacterial infections in healthcare settings, and distinct antibiotic patterns have been documented depending on the source (15).

Available literature reports indicate that there are high and worrying levels of resistance to commonly used antimicrobial drugs for treating UTIs in pregnancy, which is becoming an increasing cause for concern, especially in low- or middle-income countries (LMICs) (16).

In Somalia, empirical treatment is usually used in place of routine UTI culture and antimicrobial susceptibility testing. This could encourage the excessive use of antibiotics and the emergence of microbial strains that are resistant to them (17). In Somalia, there are not many articles expressing any alarming issues, whether it is virulence, genotypes, or antimicrobials. Moreover, there are little or no published research results on the prevalence of *E. coli* and the antimicrobial susceptibility pattern in the country. Hence, this investigation aimed to assess the prevalence and antimicrobial susceptibility profile of uropathogenic *E. coli* and its associated factors among pregnant women attending the maternity department of Dr. Sumait Hospital, Mogadishu, Somalia.

## 2 Methods and materials

### 2.1 Design of the study and its setting

A cross-sectional approach was used to study pregnant women attending the antenatal care department at Dr. Sumait Hospital in Mogadishu, Somalia, between August 2022 and December 2022.

### 2.2 Study participants and sampling

Employing consecutive convenient sampling, a cohort of 220 pregnant women meeting the inclusion criteria willingly enrolled in the study to assess the prevalence of *E. coli*. The isolates examined were of community-acquired origin. Pregnant women who declined participation and those who had undergone antimicrobial therapy within the past 7 days were exempted from the study.

### 2.3 Sample size calculation

The number of participants was determined based on the Cochrane formula. A reported prevalence of 17.3% for *E. coli* was used, with the assumption of a 95% confidence level and a desired absolute precision of 5% (18).


$$n=\left(Z\left(1-\alpha /2\right)\right)2\;p\;\left(1-p\right)/d2\text{.}$$

A sample size of 220 pregnant women was achieved.

### 2.4 Data collection tools

#### 2.4.1 Questionnaire

The pregnant study participants were interviewed using a structured questionnaire. The questionnaire comprises two sections: sociodemographic data of the respondents, including marital status, age, monthly income, and level of education; and questions regarding the potential risk factors for UTIs, including clinical data.

#### 2.4.2 Sample collection and processing

The patients voided a modest volume of urine, and midstream urine samples (220) were then collected into a sterile container. All the samples were immediately examined within an hour after collection.

Then, using a disposable loop and following conventional culture protocols, 10 μL of the urine sample was inoculated on blood and MacConkey agar (Oxoid, United Kingdom) by streaking.

Bacterial growth was then discovered after the plates had been cultured at 37°C overnight. The examination of growth and assessment was based on a growth rate of 10^5^ CFU/mL of *E. coli*. The bacterial isolates were Gram-stained; biochemical characterization was conducted, including citrate, triple sugar iron, indole, methyl red, urea agar, and motility tests, which were utilized to detect *E. coli.*

#### 2.4.3 Antimicrobial susceptibility testing

Antimicrobial resistance was assessed using the Kirby–Bauer disk diffusion method according to the Clinical and Laboratory Standards Institute protocol (19). Colonies of the bacteria were resuspended in a normal saline solution to obtain a turbidity of 0.5 McFarland standard before being applied to cover the surface of a Mueller–Hinton agar plate. The bacterial isolate was tested against a panel of 13 antimicrobials (amikacin (10 μg), nitrofurantoin (300 μg), meropenem (10 μg), gentamicin (10 μg), amoxicillin/clavulanic (20/10 μg), cefoxitin (30 μg), ciprofloxacin (10 μg), ceftazidime (30 μg), ceftriaxone (30 μg), nalidixic acid (30 μg), trimethoprim/sulfamethoxazole (25 μg, 1.25/23.75 μg), and ampicillin (10 μg)). After embedding the Mueller–Hinton agar plate with the antibiotic disks, the plate was then incubated at 37°C for 18–24 h. Resistance was measured based on the zone of growth inhibition according to the CLSI guidelines.

### 2.5 Statistical analysis and management tools

The raw data were tested for accuracy, consistency, and comprehensiveness. After that, the information was cleaned, coded, entered, and examined using SPSS version 26. A descriptive analysis was condensed using frequencies and percentages. To identify any independent variables connected to *E. coli*, a binary logistic regression model and odds ratio (OR) analysis were also used. Significance was determined when the *p*-value was ≤0.05.

## 3 Results

This study involved a total of 220 expectant women. Most (58.6%) study participants were 21–30 years old. A total of 85.9% were married, 40.5% had no formal education, 71.4% were unemployed, and 56.8% had a monthly income below US$200 (Table 1).

**Table 1:** Socioeconomic and demographic attributes of pregnant women.

	Frequency	Percentage
Age group		
< 20 years	18	8.2
21–30 years	129	58.6
31–40 years	65	29.5
41–50 years	8	3.7
Level of education		
No formal education	89	40.5
Primary	23	10.5
Secondary	73	33.1
University	35	15.9
Marital status		
Married	189	85.9
Not marital in relation	31	14.1
Occupational status		
Employed	63	28.6
Unemployed	157	71.4
Family income		
< 200 $	125	56.8
≥ 200 $	95	43.2

With respect to the age of gestation, most of the women (62.7%) were found to be in their third trimester. Similarly, 55.9% were primigravida, 59.6% had no history of abortion, and 95% had no history of diabetes. Similarly, according to the study, 71.9% of the participants had previously experienced UTI, of whom 51.4% presented urinary tract infection symptoms (Table 2).

**Table 2:** Obstetric and clinical characteristics among pregnant women attending Dr. Sumait Hospital.

	Frequency	Percentage
Gestational age		
First trimester	37	16.9
Second trimester	45	20.4
Third trimester	138	62.7
Gravidity		
Multigravida	97	44.1
Primigravida	123	55.9
History of abortion		
Yes	89	40.4
No	131	59.6
History of diabetic mellitus		
Yes	11	5
No	209	95
History of UTI		
Yes	158	71.9
No	62	28.1
Presence of UTI symptoms		
Yes	108	49.1
No	112	50.9

**Figure 1:**
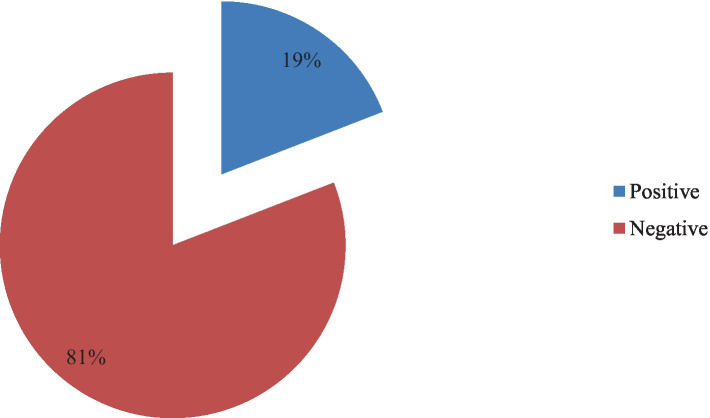
Prevalence of *E. coli* infection among pregnant women.

Concerning the occurrence of *E. coli* 19.1% of the urine specimen collected from the pregnant women were found to be positive for an *E. coli* infection, as depicted in Figure 1.

### 3.1 Prevalence of bacterial uropathogens and their distributions among pregnant women

**Figure 2:**
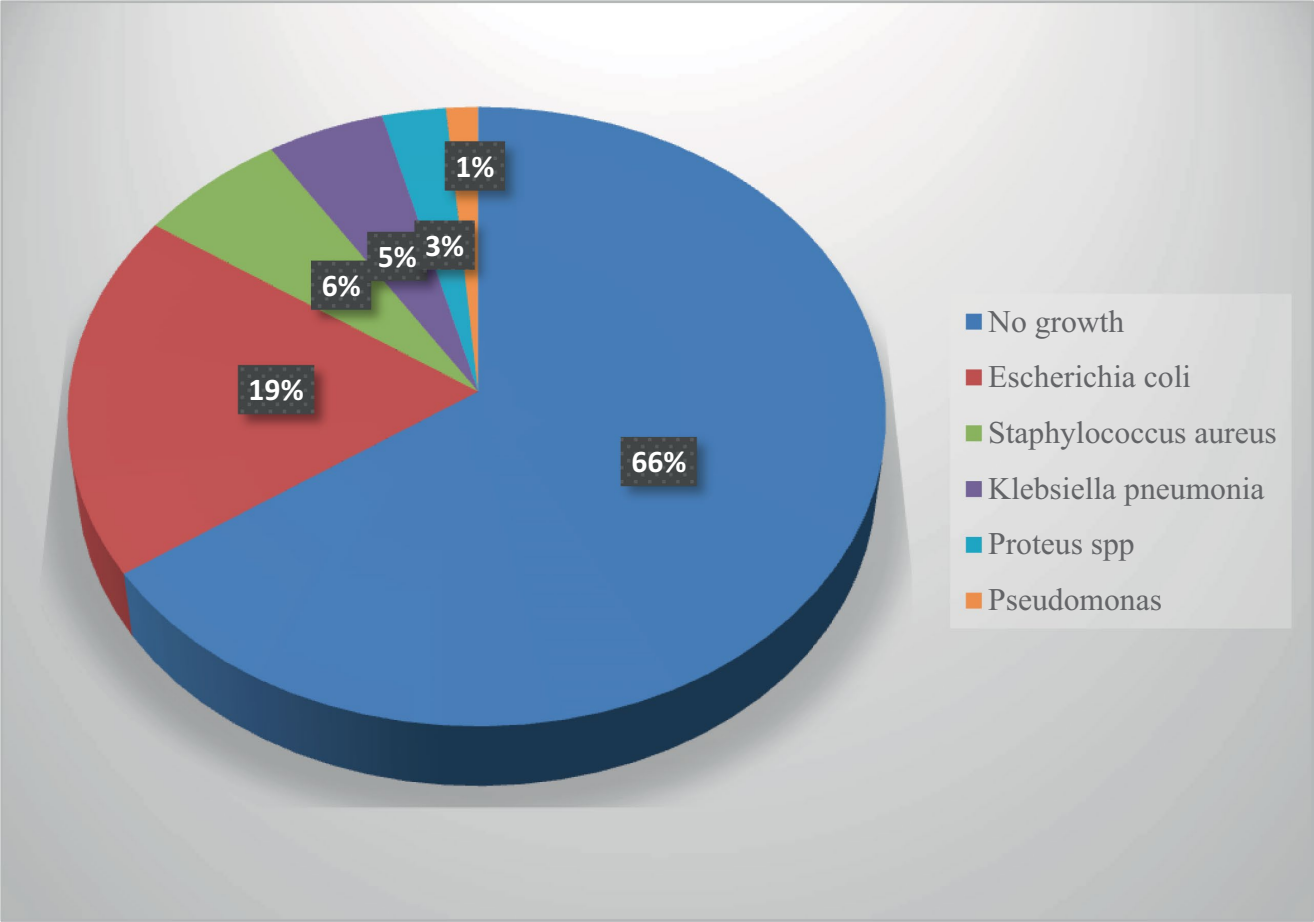
Prevalence and distribution of uropathogenic bacteria.

Out of the entire 220 analyzed urine specimens, 76 (34.5%) tested positive for significant bacteriuria. The bulk of the isolated 62 (28.1%) were the majority, constituting Gram-negative specimens, while the remaining 14 (6.3%) were Gram-positive. The most prevalent isolate was *E. coli,* 42 (19.1%) subsequent to *S. aureus* 14 (6.3%), *K. pneumoniae* 11 (5%), *Proteus* spp. 6 (2.7%), and *Pseudomonas* 3 (1.3%) (Figure 2).

The antibiotic susceptibility testing against *E. coli* isolates (Table 3) showed more susceptibility to amikacin (100%) followed by nitrofurantoin (85.7%), meropenem (83.3%), amoxicillin/clavulanic (83.3%), gentamicin (76.2%), cefoxitin (57.1%), ciprofloxacin (31%), ceftazidime (23.8%), ceftriaxone (21.4%), nalidixic acid (19.1%), trimethoprim + sulfamethoxazole (9.5%), and ampicillin (7.1%) (Table 3).

**Table 3:** Antimicrobial susceptibility of *E. coli* isolates to the common antibiotics.

Antibiotics	Interpretation		
	Susceptible	Resistance	Intermediate
Amikacin	42 (100)	0 (0.0)	0 (0.0)
Nitrofurantoin	36 (85.7)	6 (14.3)	0 (0.0)
Meropenem	35 (83.3)	7 (16.7)	0 (0.0)
Gentamicin	32 (76.2)	10 (23.8)	0 (0.0)
Cefoxitin	24 (57.1)	15 (35.7)	3 (7.1)
Amoxicillin/clavulanic	35 (83.3)	7 (16.7)	0 (0.0)
Ciprofloxacin	13 (31)	27 (64.3)	2 (4.8)
Ceftazidime	10 (23.8)	32 (76.2)	0 (0.0)
Ceftriaxone	9 (21.4)	33 (78.2)	0 (0.0)
Nalidixic acid	8 (19.1)	34 (80.9)	0 (0.0)
Sulfamethoxazole	4 (9.5)	38 (90.5)	0 (0.0)
Ampicillin	3 (7.1)	39 (92.9)	0 (0.0)

### 3.2 Factors related to *Escherichia coli* infection among pregnant mothers

The study also revealed that family income, gestational age, previous history of UTIs, and presence of symptoms of UTIs demonstrated a significant association with *E. coli* infection among pregnant women. Regarding family income, pregnant women who were from a family with a monthly income below US$200 during the study period were 2.5 times more likely to be positive for *E. coli* infection in contrast to those who had a higher family income [OR = 2.5, 95%CI:1.19–5.32, *p* < 0.015]. Similarly, expectant mothers who were in the first and second trimesters during the study period were 6.2 and 2.6 times more probable to be positive for *E. coli* infection compared to those who were in the third trimester, respectively [OR = 6.2, 95% CI:2.58–14.5, *p* < 0.050] and [OR = 2.6, 95%CI;1.11–6.30, *p* < 0.050]. Concerning the history of UTIs, expectant mothers with a UTI history were 4.5 times more likely to experience an *E. coli* infection compared to those without a UTI history [OR = 4.5, 95%CI:1.56–13.47, *p* < 0.050]. Moreover, the presence of UTI symptoms shows that expectant mothers who have symptoms of UTI are 2.4 times more likely to be positive for *E. coli* infection compared to those who have no symptoms [OR = 2.4, 95%CI: 1.16–5.20, *p* < 0.050]. On the other hand, the study did not find any statistically significant association between age, educational level, occupational status, gravidity, history of abortion, history of diabetic mellitus, and *E. coli* infection (Table 4).

**Table 4:** Factors linked to uropathogenic *E. coli* among pregnant women.

Characteristics	*N* %	*Escherichia coli* test results		OR (95% CI)	*p*-value
		Present *N*(%)	Absent *N*(%)		
Age in years					
< 20	18 (8.2)	5(11.9)	13(7.3)	Ref	
21–30	129(58.7)	21(50)	108(60.7)	0.50(0.16–1.56)	0.23
31–40	65(29.5)	13(31)	52(29.2)	0.65(0.19–2.15)	0.48
41–50	8(3.6)	3(7.1)	5(2.8)	1.56(0.26–9.10)	0.62
Educational level					
Illiterate	89(40.5)	12(28.5)	77(43.2)	0.90 (0.30–2.88)	0.90
Primary	23(10.4)	8(19)	15(8.4)	3.25(0.89–11.48)	0.07
Secondary	73(33.1)	17(40.5)	56(31.5)	1.82(0.61–5.42)	0.28
University	35(16)	5(12)	30(16.9)	Ref	
Occupational status					
Employed	63(28.6)	11(26.2)	58(24.7)	0.73 (0.34–1.56)	0.42
Unemployed	157(71.4)	31(73.8)	120(75.3)	Ref	
Family income					
< 200 $	125(56.8)	31(73.8)	94(52.8)	2.51(1.19–5.32)	0.015^*^
≥ 200 $	95(43.2)	11(26.2)	84(47.2)	Ref	
Gestational age					
First trimester	37(16.9)	16(38)	21(11.8)	6.24(2.68–14.5)	0.000^*^
Second trimester	45(20.4)	11(26.2)	34(19.1)	2.65(1.11–6.30)	0.027^*^
Third trimester	138(62.7)	15(35.8)	123(69.1)	Ref	
Gravidity					
Multigravida	97(44.1)	17 (40.5)	80(44.9)	0.48 (1.13–1.00)	0.053
Primigravida	123(55.9)	25(59.5)	98(55.1)	Ref	
History of abortion					
Yes	89(40.4)	19(45.2)	70(39.3)	1.29(0.64–2.51)	0.483
No	131(59.6)	23(54.8)	108(60.7)	Ref	
History of diabetic mellitus					
Yes	11(5.0)	3(7.1)	8(4.4)	1.63(0.41–6.44)	0.482
No	209(95.0)	39(92.9)	170(95.6)	Ref	
History of UTIs					
Yes	158(71.9)	38(90.4)	120(67.4)	4.59(1.56–13.47)	0.0055^*^
No	62(28.1)	4(9.6)	58(32.6)	Ref	
Presence of UTI symptoms					
Yes	126 (57.3)	31(73.8)	95(53.4)	2.46(1.16–5.20)	0.018*
No	94 (42.7)	11 (26.2)	83(46.6)	Ref	

*Indicate statistical significance.

The study did not find any statistically significant differences regarding family monthly income, gestational age, and history of UTI in women with *E. coli* infection and in women with infection due to other uropathogens (Table 5).

**Table 5:** Potential risk factors associated with uropathogenic *E. coli* and women with other uropathogenic infections.

Characteristics	*N* (%)	Uropathogenic status		95% OR	*p*-value
		E-coli infection	Other infection		
Income					
< 200 $	51 (67.1)	28 (66.7)	23(67.7)	0.95(0.36–2.51)	0.9279
≥ 200 $	25(32.9)	14 (33.3)	11(32.3)	Ref	
Gestational age					
First trimester	32(42.2)	19(45.2)	13(38.2)	1.78(0.57–5.52)	0.3137
Second trimester	24(31.5)	14(33.4)	10(29.4)	1.71(0.51–5.66)	0.3794
Third trimester	20(26.3)	9(21.4)	11(32.4)	Ref	
UTI history					
Yes	47(42.1)	31(73.9)	20(58.9)	1.92(0.74–5.20)	0.1695
No	29(57.9)	11(26.1)	14(41.1)	Ref	

As seen in Table 6, there is a statistically significant difference between women with *E. coli* infection and those with negative urine culture reports regarding their monthly income and history of UTI (*p* = 0.0014 and *p* = 0.007), respectively. However, there was no significant association between gestational age and *E. coli* infection between the groups.

**Table 6:** Potential risk factors associated with uropathogenic *E. coli* and women without other uropathogenic infections.

Characteristics	*N* (%)	Uropathogenic status		95% OR	*p*-value
		E-coli infection	Negative uropathogenic		
Income					
< 200 $	79(54.9)	32(76.1)	47(46)	3.74(1.66–8.41)	0.0014*
≥ 200 $	65(45.1)	10(23.9)	55(54)	Ref	
Gestational age					
First trimester	85(59)	31(73.9)	54(53)	2.29(0.61–8.77)	0.2240
Second trimester	44(30.6)	8(19)	36(35.2)	0.88(0.20–3.90)	0.8760
Third trimester	15(10.4)	3(7.1)	12(11.8)	Ref	
UTI history					
Yes	84(58.3)	34(81)	50(49)	4.42(1.86–10.47)	0.007*
No	60(41.7)	8(19)	52(51)	Ref	

*Indicate statistical significance.

## 4 Discussion

Urinary tract infections are highly prevalent during pregnancy, with *E. coli* being the most common pathogen among expectant mothers. The anatomical and physiological alterations in the body during pregnancy can increase women’s vulnerability to developing UTIs (20). This study found that 19% (42/220) of the pregnant women were positive for *Escherichia coli*. A higher prevalence was reported in Kenya at 23.5% (21), southeast Ethiopia at 27.3% (22), and south-western Uganda at 28.78% (23). The variation may result from local social norms, environmental factors, personal hygiene expectations, and healthcare utilization patterns.

Regarding sociodemographic characteristics, it has been found that the monthly income of pregnant women below US$200 was notably associated with an *E. coli* infection. It has been revealed that expectant mothers who had a low monthly income were twice as likely to have UPEC compared to those who had a higher income. Similar study findings were reported from northern Ethiopia (24). This could result from the relationship between low socioeconomic status and poor nutrition and immunity, especially in pregnant women.

According to the gestational period, pregnant women who were first and second trimesters pregnant during the study period were 6.2 and 2.6 times more likely to get an *E. coli* infection, respectively compared to their third-trimester counterparts. Studies conducted in various locations yielded similar results in Saudi Arabia and Western and Northern Ethiopia (11, 24, 25). As argued by Tadesse et al. (24), this could be the result of UTIs in pregnant women, which usually initiate approximately at week six, reaching its peak between weeks 22 and 24. This is attributed to factors such as urethral dilation, heightened bladder volume, diminished bladder, and urethral tone, all of which foster bacterial proliferation in the urine.

Regarding the presence of UTI symptoms, expectant mothers with prior symptoms of UTIs exhibited a 2.4 times greater probability of developing a positive *E. coli* infection compared to those who had no symptoms. A similar study finding was reported in Khartoum, Sudan (26). This may be because *E. coli* is strongly linked to the typical symptoms of infection.

Moreover, the study revealed that pregnant women with a prior history of UTIs had a 4.5-fold higher likelihood of *E. coli* infection compared to individuals without a history of UTIs. This was similar to other studies from Libya, Egypt, and Nigeria (27–29). This association might be explained by the presence of strains resistant to antibiotics from previous infections.

In our study, no statistically significant correlation was observed between the prevalence of *E. coli* and age, educational attainment, employment status, gravidity, history of abortion, or history of diabetes mellitus. Studies conducted in Bangladesh and Nigeria revealed similar findings (30, 31). However, these studies reported that *E. coli* has a significant association with the mother’s gestational age. However, according to the comorbidities, previous studies identified that there is a significant association between having diabetes mellitus and chronic kidney disease and the risk of developing UTIs due to their altered immunological integrity (32).

As per the study findings, no distinctions were observed between uropathogenic *E. coli* and other uropathogenic infections concerning potential risk factors such as income, gestational age, and history of urinary tract infections. Comparable results were observed in studies conducted in both Eastern and Southeast Ethiopia (22, 33). On the other hand, it was observed that women from low-income families and those with a history of UTIs were 3.7 and 4.4 times more likely to contract an *E. coli* infection compared to their counterparts. A similar result was reported by a study conducted in Central Ethiopia (17). This might suggest that individuals with a lower income experience diminished socioeconomic status, leading to inadequate nutrition and weakened immunity, thereby elevating the likelihood of uropathogenic infections. Additionally, a prior history of UTIs during pregnancy, coupled with the persistence of antibiotic-resistant strains from the earlier infection, could contribute to the recurrence of such infections.

According to the study’s analysis of antibiotic susceptibility patterns, *E. coli* was most susceptible to amikacin (100%), followed by nitrofurantoin (85.7%), meropenem (83.3%), amoxicillin/clavulanic (83.3%), and gentamicin (76.2%). Similar results were observed in studies conducted in South Africa and Ethiopia (34–36). However, *E. coli* were resistant to nalidixic acid (80.9%), sulfamethoxazole (90.5%), and ampicillin (92.9%) This aligned with research conducted in Nairobi, Kenya (37).

According to the current study, uropathogenic *E. coli* bacteria were exceedingly resistant to third-generation cephalosporin antibiotics. Similarly, the World Health Organization (WHO) also observed resistance to third-generation cephalosporins in 68% of *E. coli* isolates and 81% of Klebsiella isolates (38, 39). Additional studies related to multi-drug resistance in uropathogenic *E. coli* bacteria have also been described (16, 40). The present investigation indicated that, regarding simple and complex bacteriuria, carbapenems are preferable to cephalosporins. The most prescribed antibiotics are easily accessible at nearby drugstores in Somalia, and people can buy and utilize them without needing a prescription, potentially hastening the onset of drug resistance.

## 5 Conclusion

The prevalence of *E. coli* was 19%. Monthly income, gestational period, previous experience of UTIs, and existence of symptoms of UTIs were associated with *E. coli* in antibiotic sensitivity patterns. The main antibiotics that proved effective against *E. coli* isolated from urine samples in pregnant women were amikacin, nitrofurantoin, and meropenem. It could be recommended that pregnant women in antenatal care have routine culture and antimicrobial susceptibility tests to prevent resistance and complications in both the expectant mother and the unborn child.

## Data availability statement

The raw data supporting the conclusions of this article will be made available by the authors, without undue reservation.

## Ethics statement

The studies involving humans were approved by SIMAD University’s Human Research Ethics Review Board. The studies were conducted in accordance with the local legislation and institutional requirements. Written informed consent for participation in this study was provided by the participants’ legal guardians/next of kin.

## Author contributions

FM: conceptualization. MA: methodology and data curation. HMD: data collection. JM: formal data analysis. HAD: writing original draft preparation. FM, HAD, JM, MA, and HMD: writing—review and editing. All authors contributed to the article and approved the submitted version.
